# Relationship between histological tumor margins and magnetic resonance imaging signal intensities in brain neoplasia of dogs

**DOI:** 10.1111/jvim.16431

**Published:** 2022-04-30

**Authors:** Philippa J. Johnson, Benjamin C. Rivard, Jonathan H. Wood, Mattisen L. DiRubio, Joshua G. Henry, Andrew D. Miller

**Affiliations:** ^1^ Department of Clinical Sciences Cornell College of Veterinary Medicine, Cornell University Ithaca New York USA; ^2^ Department of Biomedical Sciences, Section of Anatomic Pathology Cornell College of Veterinary Medicine, Cornell University Ithaca New York USA

**Keywords:** glioma, histiocytic sarcoma, meningioma, oligodendroglioma, registration

## Abstract

**Background:**

Intracranial neoplasia is relatively common in dogs and stereotactic radiotherapy, surgical debulking, or both, are the most successful treatment approaches. A key component of treatment planning involves delineating tumor margin on magnetic resonance imaging (MRI) examinations. How MRI signal intensity alterations relate to histological tumor margins is unknown.

**Objectives:**

Directly compare histological brain sections to MRI sequence images and determine which sequence alteration best correlates with tumor margins.

**Animals:**

Five dogs with glioma, 4 dogs with histiocytic sarcoma, and 3 dogs with meningioma.

**Methods:**

Retrospective cohort study. Histological brain sections were registered to in vivo MRI scan images obtained within 7 days of necropsy. Margins of signal intensity alterations (T2‐weighted, fluid‐attenuating inversion recovery [FLAIR], T1‐weighted and contrast enhancement) were compared directly to solid tumor and surgical margins identified on histology. Jacquard similarity metrics (JSM) and cross‐sectional areas were calculated.

**Results:**

In glioma cases, margins drawn around T2‐weighted hyperintensity were most similar to surgical margins (JSM, 0.66 ± 0.17) when compared to other sequences. In both meningioma (JSM, 0.57 ± 0.21) and histiocytic sarcoma (JSM, 0.75 ± 0.11) margins of contrast enhancement were most similar to surgical margins.

**Conclusions and Clinical Importance:**

Signal intensities correspond to tumor margins for different tumor types and facilitate surgical and radiation therapy planning using MRI images.

**Abbreviations**
CSA: cross‐sectional area
DICOM: Digital Imaging and Communications in Medicine
FLAIR: fluid attenuating inversion recovery
JPEG: Joint Photographic Experts Group
JSM: Jaccard similarity metric
PACs: picture archiving system
TIFF: tag image file format

## 1 INTRODUCTION

Intracranial neoplasia is relatively common dogs, with a recent retrospective study showing an overall prevalence of 4.5%.^1^ The most common primary intracranial neoplasms of dogs include meningioma, representing nearly 50%, and glioma representing 30%‐40% of all primary brain tumors.^1^ The remaining 10%‐20% consist of a variety of diagnoses including histiocytic sarcoma.^1^ Currently, the prognosis of dogs diagnosed with brain tumors remains poor despite use of multimodal treatment.^2^, ^3^, ^4^

Stereotactic radiotherapy, surgical excision or both or debulking are the most successful treatment approaches for intra‐ and extra‐axial intracranial tumors in the dog.^5^ Determining precise tumor margins and target volumes are prerequisites for successful surgical management and radiotherapy planning, with magnetic resonance imaging (MRI) being the modality of choice to resolve tumor margins because of its high soft tissue contrast resolution.^6^, ^7^ Imaging features are recognized for the most common intracranial neoplasms in dogs, with MRI providing information about tumors according to their signal characteristics, as observed on the sequences performed.^8^, ^9^ Routine brain MRI examinations include fluid‐attenuating inversion recovery (FLAIR), T2‐weighted, T1‐weighted, and post‐contrast sequences. From these sequences, information on tumor structure, margination, vascularization, hydration, as well as peritumoral edema and inflammation is assessed. To date, however, no histopathological confirmation about the meaning of these alterations in signal characteristics has been made for different tumor types in dogs. More importantly, for radiation and surgical planning, the correlation between histopathologic tumor margins and in vivo MRI signal characteristics is not well understood.^7^

In human medical neuro‐oncology, direct comparisons between whole brain histopathology and in‐vivo imaging is not possible because of the often extended time between clinical in vivo imaging and death. Necropsy MRI results have been directly compared to whole brain histopathology.^10^ However, the applicability of these results to the evaluation of mass lesions on in vivo MRI is limited. Targeted biopsy of brain regions has provided valuable information about the presence or absence of tumor in areas of signal intensity alteration on MRI, but the technique is limited when compared to whole brain histopathology.^11^, ^12^, ^13^ In veterinary patients, euthanasia often is elected within a short time period after imaging diagnosis, providing the ability to obtain whole brain histopathology within days of in vivo imaging.

Our aim was to determine whether MRI characteristics of 3 common intracranial neoplasms can be correlated with histopathologic findings on necropsy that would facilitate decision making and clinical management for affected dogs. We investigated the correlation between signal intensity alterations identified on routine MRI sequences with tumor and surgical margins identified on histopathology of the corresponding region of brain and tumor. Accurate evaluation of tumor margins would allow for more effective surgical planning, decreasing the chance of incomplete excision, and would be helpful in determining optimal radiation treatment volumes to include all neoplastic elements, while sparing unaffected adjacent brain parenchyma.

## 2 MATERIALS AND METHODS

### 2.1 Subjects

The medical recording system and anatomic pathology archives were searched for affected dogs at the Cornell University Hospital for Animals and the New York State Animal Diagnostic Health Center, respectively. Inclusion criteria for the study were as follows: the dog had a diagnosis of a brain tumor, the dog had undergone both 1.5‐T MRI and necropsy examination within 7 days of each other, the MRI study performed was of diagnostic quality without clinically relevant artifacts and had comparative transverse T2‐weighted, FLAIR, T1‐weighted and post‐contrast sequences, and the histopathology obtained of the brain included representative, high quality, transverse plane sections. Only tumor types that had at least 3 cases that met the inclusion criteria were included in the study.

### 2.2 Dog information

The breed, sex, weight, age, interval between MRI and necropsy, tumor type and pertinent medication received were recorded for each dog.

### 2.3 MRI examination

All dogs had undergone an in vivo standardized brain protocol MRI using a 1.5‐T system (Toshiba Vantage Atlas). Sequences performed included T1‐weighted, T2‐weighted, FLAIR and T1‐weighted post contrast (MRI parameters used are presented in Table 1). At our institution, transverse MRI slices are aligned with the cerebellar peduncles, which corresponds to the way the brain is sliced for histopathology sectioning. The MRI examinations were evaluated using Horos picture archiving and communications system (PACS). Brain MRI examinations were accessed using a commercial PACS (VuePACS ver. 12.2.2; Carestream Health, Inc. Rochester, New York), and the selected transverse MRI images in T1‐weighted, T2‐weighted, FLAIR and gadolinium contrast‐enhanced T1‐weighted pulse sequences were exported into digital imaging and communications in medicine (DICOM) and joint photographic experts group (JPEG) file formats on a computer workstation. Studies were evaluated by a board‐certified radiologist (PJJ) to ensure they met the inclusion criteria.

**TABLE 1:** Documents the range of magnetic resonance imaging parameters used for the sequences included in the study.

	Echo time	Repetition time	NEX	Flip angle	Slice thickness	Matrix size	Echo train length	Phase encoding steps
FLAIR	94‐100	6700‐8100	2	90	2.7‐3.5	320‐384 × 288‐352	13‐19	286‐312
T2	90‐94	5155‐6065	2‐3	90	2.7‐3.6	480‐512 × 320‐352	13‐17	312‐403
T1	10‐12	385‐595	2‐3	90	2.7‐3.7	320‐480 × 320‐352	4	320‐412

### 2.4 Histopathology

All animals had necropsy performed within 48 hours of euthanasia and appropriate tissues were collected and fixed in 10% neutral buffered formalin before processing and sectioning for histologic analysis. All dogs were required to have at least 1 representative slide of the sectioned brain tumor. Each available slide was evaluated for quality and distortion. Because the study required transformation of histopathology slides to match MRI images, slides that exhibited excessive processing artifacts such as tearing, folding or shearing were excluded from analysis. All histology slides were scanned using a Ventana DP 200 slide scanner and high‐resolution tag image file format (TIFF)—digitized brain histopathology slides were exported to the same workstation in native uncompressed format. Slides were evaluated by a board‐certified veterinary anatomic pathologist (ADM) to ensure they met the inclusion criteria.

### 2.5 Image transformation and registration

**FIGURE 1:**
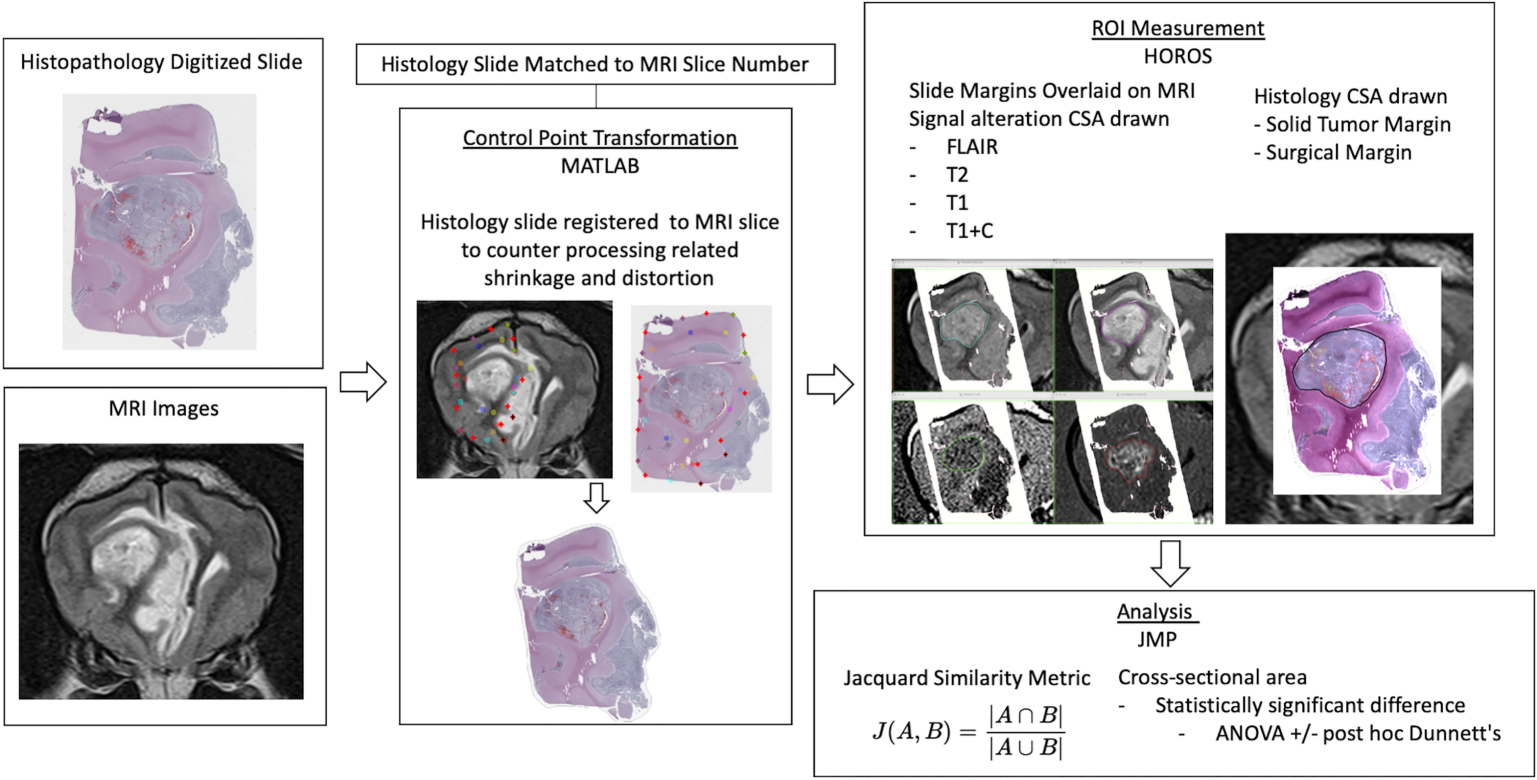
Flow chart depicting the registration, measurements, and analysis techniques used in the study

An overview of the methodology of the study is presented in a flow chart in Figure 1. Each MRI examination was reviewed together by a board‐certified veterinary anatomic pathologist (ADM), board‐certified veterinary radiologist (PJJ), and radiology resident (BCR) to match the transverse image that corresponded most closely with the representative histology sections of the brain available for that case. For each MRI image and histology scan pair, matched control points were determined by consensus between the radiologist and anatomic pathologist. These points were overlaid on the corresponding MRI and histology images, utilizing identifiable anatomic landmarks including blood vessels, sulci, gyri, gray or white matter definition, and the ventricular system. Abnormal MRI image intensity borders and histologic tumor margins were avoided for control point selection whenever possible to prevent bias in image transformation. The number of control points ranged from 14 to 40 for each transformation. The number of points placed corresponded with the number of sites where normal anatomic features were present on both MRI and the histopathology image.

The JPEG and DICOM MRI images and TIFF brain histology scans were imported into a commercial programming and numeric computing platform (MATLAB R2021a, MathWorks, Inc. Natick, Massachusetts) with feature‐specific image processing add‐ins (Computer Vision Toolbox, Image Processing Toolbox). For each MRI image and histology scan pair, an image transformation script was encoded and the processing algorithm was initiated by importing the histology scan and reorienting it using horizontal or vertical flip and angular rotation of the image to approximate the orientation of the MRI image. The corresponding T2w MRI JPEG (“original image”) was imported and opened in a graphical user interface where the previously identified control points were manually assigned by mouse‐click, creating X,Y coordinate pairs. In similar fashion, the native histology scan (“warp image”) was opened in a graphical user interface, where the previously identified control points were manually assigned by mouse‐click, creating X,Y coordinate pairs. A local‐weighted mean image transformation (with n = number of control point pairs) was performed on the warp image utilizing the matched pairs of X,Y coordinates and the built‐in image transformation algorithm of the specialized add‐in software. The transformed histology scan was displayed within MATLAB, and was saved in both JPEG and TIFF formats. A fusion image of the transformed histology scan and the original MRI image also was displayed and saved in JPEG and TIFF formats. This process was repeated identically for all images, with the exception of the number of control point pairs used and the orientation changes made to the original histopathology slide scans.

### 2.6 MRI and histology image measurements

The transformed histology image was re‐evaluated and compared to the original histology slide. Using histopathologic features from the original histopathology slide, boundaries were marked on the transformed image to document the following margins:

1. Surgical margin: the boundary between normal brain tissue and diseased tissue (ie, any tissue that contained tumor cells or inflammation).
2. Solid tumor margin: the margin where solid tumor ended (ie, did not include areas where tumor or inflammatory cells infiltrated the adjacent brain matter).

In some cases, where no infiltration or inflammation extended into the gray or white matter from the solid tumor, the solid tumor margin and surgical margins were the same. Sites of obvious peri‐tumoral edema were marked with annotations.

**FIGURE 2:**
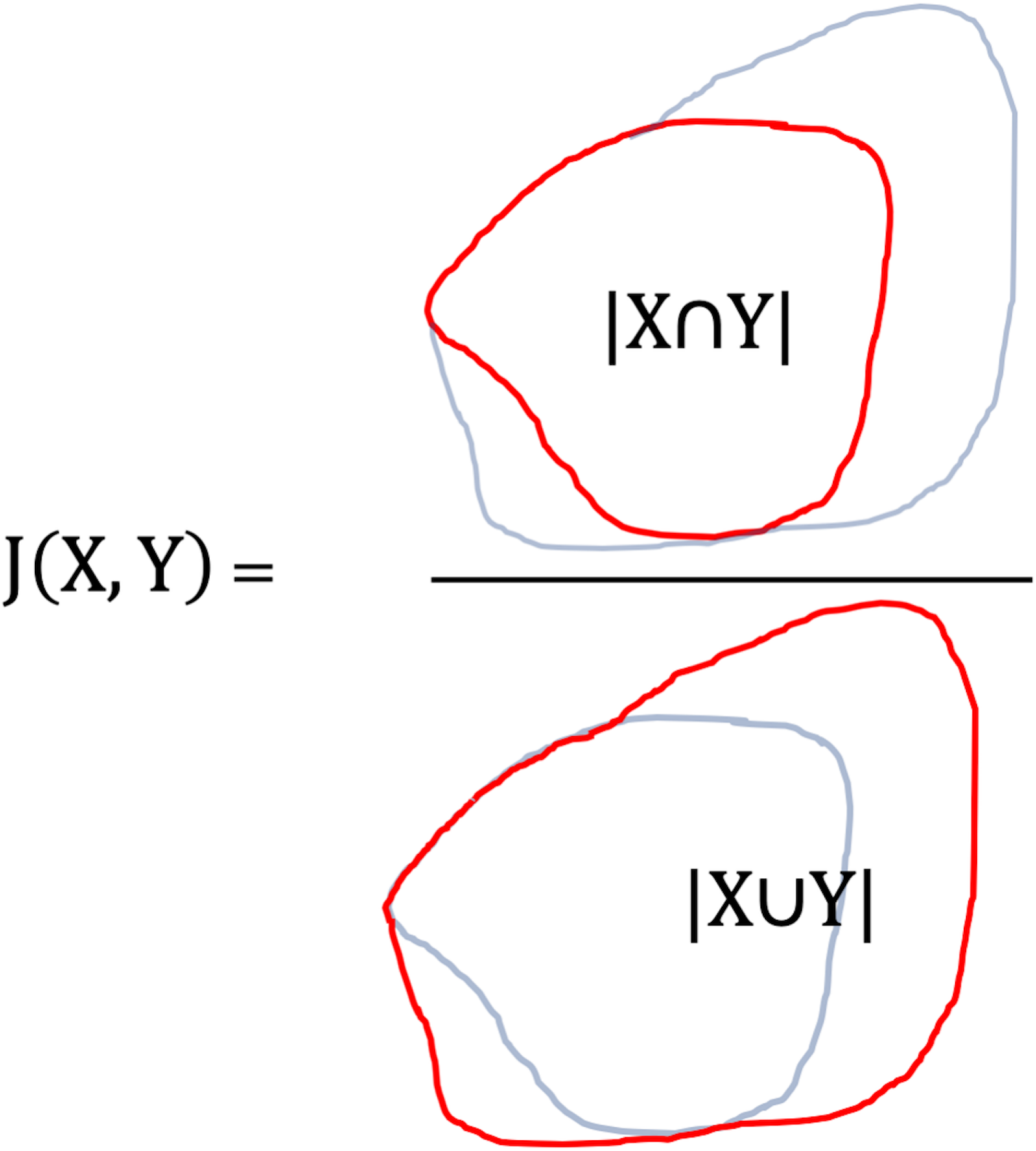
Calculation of the Jaccard similarity metric using 2 regions of interest. The overlap area was divided by the area of both regions of interest

The transformed histology and MRI images were imported into Horos (v3.3.6, www.horosproject.org) 64‐bit medical image viewing software. Transformed histologic images were fused to each MRI sequence, manually positioned and then thresholded to show only the slide boundary. The margin of signal intensity alterations present within the slide boundary for each sequence then was drawn using the pencil tool 3 times by a single observer and the average calculated. Signal intensity changes were compared to the adjacent gray or white matter, and included any hyperintensity on T2‐weighted and FLAIR sequences, hypointensity on T1‐weighted sequences and contrast‐enhancement on post‐contrast series. The fused histologic image then was windowed to make the brain tissue visible and regions of interest (ROIs) were drawn around the solid tumor and surgical margins. A representative ROI was saved and the average cross‐sectional area (CSA) recorded. To assess for similarity between histopathology and MRI‐based ROIs, images of the ROIs were obtained and imported into online software that allowed for areas to be calculated (sketchandcalc.com). The area of ROI overlaps and the area of both ROIs combined were measured, and the Jaccard similarity metric (JSM) calculated for each sequence (FLAIR, T2‐weighted, T1‐weighted, and T1‐weighted with contrast) and either solid tumor or surgical margins. The JSM is a measure of overlap between ROIs and ranges from 0 (no agreement) to 1 (complete agreement). It is calculated using the following equation: J(A, B) = |A∩B|/|A∪B| (see Figure 2).

### 2.7 Statistical analysis

The data were assessed as having a normal distribution by plotting a histogram with normal quantile plot and statistically assessing for normality using the Shapiro‐Wilk W test.

To assess how well the CSA margins that associated with signal intensity changes were correlated with histologically‐defined tumor margins, we calculated a correlation coefficient for each intensity alteration in relation to surgical and solid tumor margins. For each tumor type, the CSA measured for each sequence was plotted on an X‐by‐Y plot against the CSA for both solid tumor and surgical margin, and *r* calculated. Correlations were considered weak when the coefficient was <.50, moderate for .50‐.70, and strong for >.70. This analysis allowed us to determine how CSA correlated between sequence and tumor margins but did not provide information on overlap or location of margins.

To identify similarity between sequence ROIs and histological margins, the JSM for each tumor type was plotted using a violin plot. An analysis of variance (ANOVA) was performed to identify statistically significant differences between the JSM of each sequence, and a post‐hoc Tukey's test used to show where these differences lay. These analyses allowed us to see if 1 sequence was significantly more similar to a surgical margin than other sequences.

## 3 RESULTS

### 3.1 Subjects

Twelve dogs with confirmed brain tumors that had undergone 1.5‐T MRI of the brain and necropsy within 7 days were identified. This group of dogs consisted of 5 males and 7 females with mean body weight of 21.5 kg (SD, 14.7) and mean age of 7.98 years (SD, 1.6). This group of dogs had a necropsy‐to‐MRI interval range of 0 to 7 days (mean, ±SD of 1.9 days, ±1.9). Within this cohort, 4 were confirmed to have histiocytic sarcoma, 5 glioma, and 3 meningioma. Of the 4 histiocytic sarcoma cases, 3 were multicentric and whether they originated in the central nervous system (CNS) and metastasized widely or originated viscerally and spread to the CNS could not be determined on necropsy. Summaries of each dog's signalment, interval between MRI and necropsy and confirmed tumor type are provided in Table S1.

### 3.2 Histopathology

**FIGURE 3:**
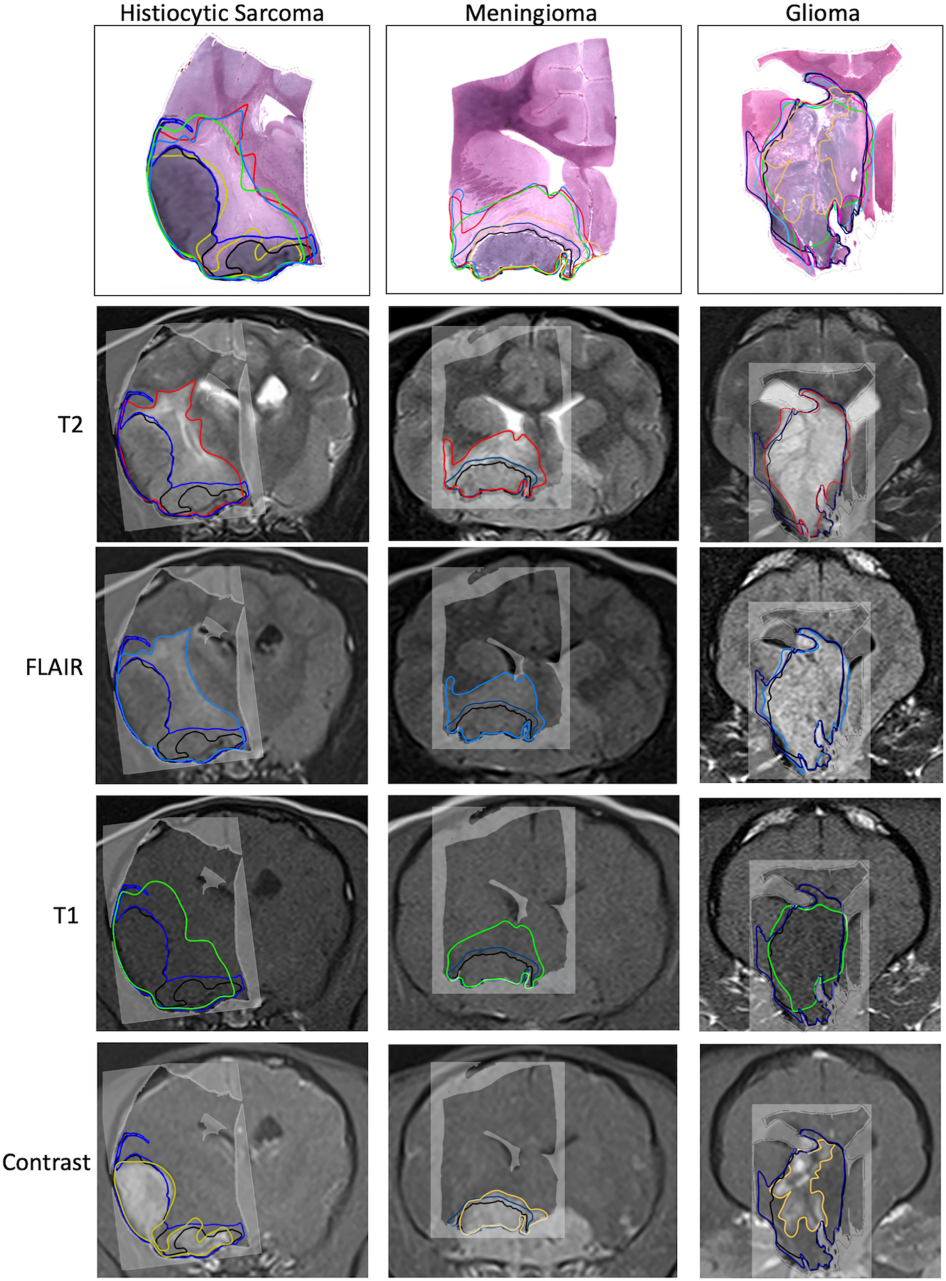
Solid tumor and surgical histology margins overlaid on T2‐weighted, fluid‐attenuating inversion recovery (FLAIR), T1‐weighted and gadolinium contrast enhancement intensity margins for histiocytic sarcoma, meningioma and glioma example cases. Line tracings depict solid tumor margin (black), surgical margin (navy blue), T2‐weighted signal intensity margin (red), FLAIR signal intensity margin (pale blue), T1‐weighted hypointensity margin (green), and contrast enhancement margins (yellow)

Histopathologic evaluation of each case identified different patterns of solid tumor and peritumoral infiltration, inflammation and edema. Within the histiocytic sarcoma group, 3 of the 4 cases had infiltration of tumor cells into adjacent brain tissue beyond the solid tumor margin, resulting in the surgical margin being larger than the solid tumor margin. All cases had edema without inflammation. Within the meningioma group, all were transitional subtypes, with 1 having atypical features. Only 1 case showed infiltration beyond the solid tumor margin, but 2 of the 3 cases exhibited a peritumoral infiltration of inflammatory cells (lymphocytes, plasma cells, and macrophages) within brain tissue. One meningioma case had edema in the adjacent neuroparenchyma. Within the glioma group, 4 were high‐grade oligodendrogliomas and 1 was a high‐grade undefined glioma. Infiltration of neoplastic glial cells into the adjacent neuroparenchyma was present in 3 cases and not identified in 2 cases. Three glioma cases had edema in the surrounding neuroparenchyma (Figure 3). Perilesional inflammation was not identified in any of the glioma cases.

### 3.3 Comparing MRI signal intensity changes to histopathological margins

#### 3.3.1 CSA measures

**FIGURE 4:**
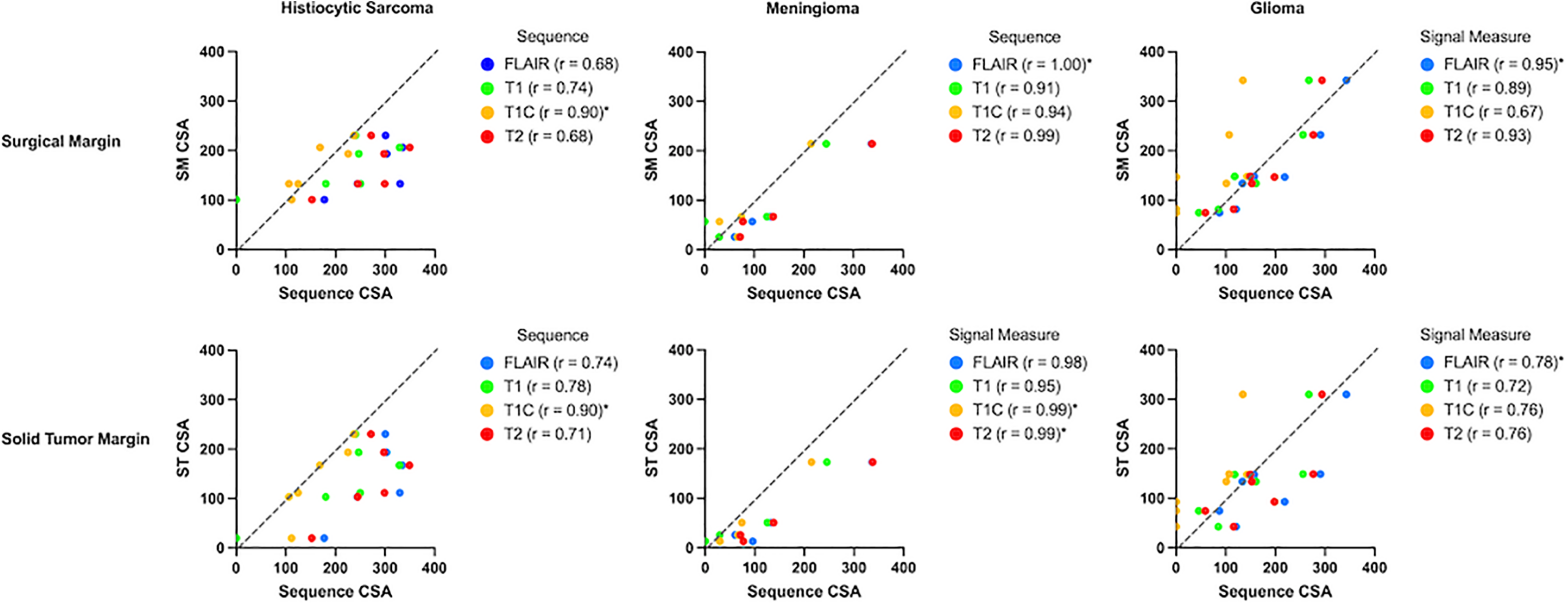
Demonstrates scatterplots of each sequence cross‐sectional area (CSA) (fluid‐attenuating inversion recovery in blue, T2‐weighted in red, T1‐weighted in green and post contrast in yellow) plotted against the measured histological margin CSA (solid tumor, surgical margin) for each tumor type

Scatterplots and associated correlation coefficients are presented in Figure 4. In histiocytic sarcoma cases the T1‐weighted post contrast series showed the highest correlation to both solid tumor and surgical margins (*r* = .90 for both). The analysis for meningioma was limited by the low case numbers, with all sequences having a very high to perfect correlation to both surgical and solid tumor margins. For gliomas, the FLAIR sequence, closely followed by the T2‐weighted sequence, had the highest correlation to both solid tumor and surgical margins (FLAIR *r* = .78 for solid tumor margin and *r* = .95 for surgical margin, and T2‐weighted *r* = .76 for solid tumor margin and *r* = .93 for surgical margin).

### 3.4 Jaccard similarity metric

**FIGURE 5:**
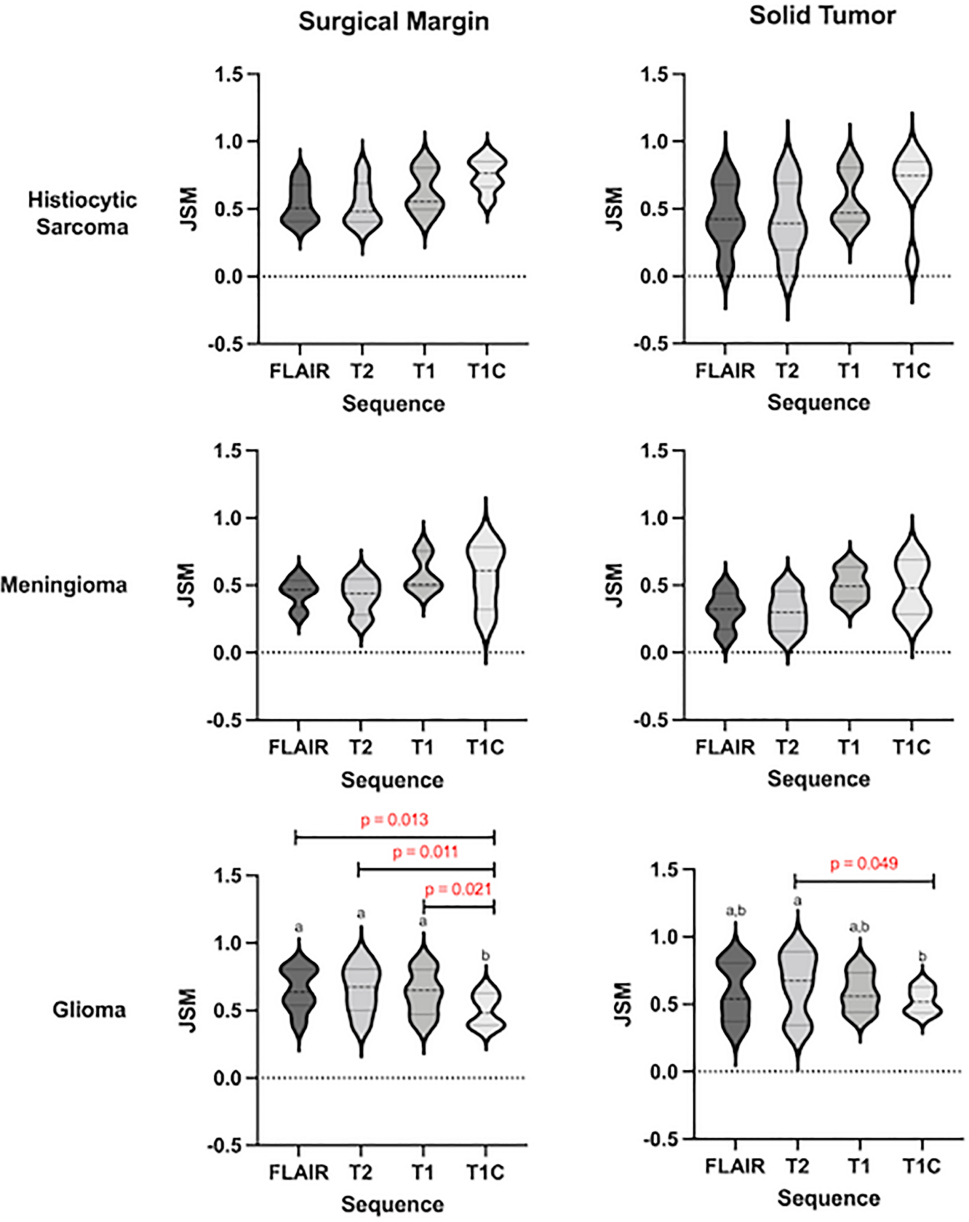
The Jacquard similarity metrics for each signal intensity alteration in relation to either surgical margin (left) and solid tumor margin (right)

The ROIs drawn around the periphery of the signal intensity changes were compared to ROIs drawn around the solid tumor and surgical margins. The JSMs, which consider how much overlap the regions contain, were calculated for each sequence and histological margin for each tumor type and are plotted in Figure 5. For histiocytic sarcoma and meningioma, the contrast sequence showed the highest similarity to the histology metrics, being most similar to the surgical margin (mean ± SD, 0.75 ± 0.11 and 0.57 ± 0.21, respectively) rather than the solid tumor margin (mean ± SD, 0.67 ± 0.26 and 0.48 ± 0.18, respectively). The differences between the signal intensity JSMs were not significantly different, when tested using ANOVA.

For the glioma group, the T2‐weighted, FLAIR and T1‐weighted sequence signal intensity margins showed the highest similarities to the histology margins, with the T2‐weighted sequence being the most similar. These sequences were more similar to surgical margins (T2‐weighted, mean ± SD, 0.66 ± 0.17; FLAIR, 0.65 ± 0.15; T1, 0.63 ± 0.16) than solid tumor margins (T2‐weighted mean ± SD, 0.64 ± 0.22; FLAIR, 0.59 ± 0.2; T1, 0.58 ± 0.14). Statistically significant differences were identified between sequences, when using a 1‐way ANOVA for surgical margins (*P* = .01) and for solid tumor margins (*P* = .04) with the contrast sequence having a significantly lower JSM than T2‐weighted sequences (*P* = .05) for solid tumor margin and being significantly lower than T2‐weighted (*P* = .01), FLAIR (*P* = .01), and T1‐weighted (*P* = .02) sequences for surgical margins.

## 4 DISCUSSION

We registered histologic sections of brain to MRI to allow for direct comparison of in vivo MRI sequence signal intensity alterations with histologically confirmed tumor margins. In doing so, we identified which sequence signal alterations had the highest correlation in CSA and exhibited the most overlap (JSM) to histologically‐ confirmed tumor margins. We found that contrast enhancement margins were most similar (had the highest JSM) to surgical margins in histiocytic sarcomas, and their CSA correlated highly to both surgical margins and solid tumor margins. In meningiomas, all sequence CSAs had similarly high correlation coefficients, but margins of contrast enhancement were most similar to surgical margins. In gliomas, both FLAIR and T2‐weighted sequence CSAs were highly correlated to surgical margins and T2‐weighted sequence margins were most similar to surgical margins. These findings improve the ability to determine how signal intensity changes correlate to neoplastic margins in these types of brain neoplasia. In terms of surgical planning, this approach allows more accurate tailoring of the surgical dose administered to the dog and avoids insufficient tumor resection or unnecessary removal of normal brain parenchyma at the tumor margin. For radiation planning, this information allows more accurate determination of tumor volume for radiation fraction dosage calculation.

The 5 cases of glioma all were graded as high grade with 4 being oligodendrogliomas and 1 undefined.^14^ The MRI appearance of gliomas has been well described but can be highly variable among glioma types and grades.^8^, ^15^, ^16^, ^17^ Histopathologically, gliomas are composed of 3 distinct regions: an inner core of solid tumor, a surrounding penumbra of infiltrating tumor cells and a coincident region of edematous parenchyma.^11^, ^18^ Being able to accurately define which of these regions correlated to different signal intensity changes is important for radiation and surgical planning as well as for monitoring tumor progression. We found that T2‐weighted, T1‐weighted and FLAIR signal changes had the highest similarity and closest correlation to surgical margins rather than to solid tumor margin. All of these sequence signal intensity changes relate to an increase in fluid content of the tissue. Our findings suggest that these signal changes highlight areas of the infiltrating neoplastic penumbra, rather than solid tumor or perilesional edema alone. This finding is consistent with what has been observed in gliomas of humans, where fluid‐rich T2‐weighted signal hyperintensity was found to include infiltrative neoplastic cells,^11^, ^12^, ^13^ and complete resection of all FLAIR hyperintensity regions results in higher survival rates.^19^ We found that contrast enhancement in the glioma cases was highly variable, as has been consistently described,^8^, ^15^, ^16^ and did not correlate with solid tumor or surgical margins, being consistently smaller in CSA. This finding indicates that margins of contrast enhancement should not be an MRI feature used for surgical or radiation planning purposes, and highlights the shortcomings of previous studies that have used contrast enhancement to represent tumor margins in gliomas.^20^, ^21^ The T2‐weighted hyperintense tissue is fluid‐rich and correlates to hypoattenuating regions on computed tomography (CT). Because the majority of radiation planning is done using CT in veterinary medicine, our findings support the use of fluid‐rich CT hypoattenuation margins for radiation planning of glioma, rather than margins of contrast enhancement.

We identified that, in both extra‐axial tumor types (meningiomas and histiocytic sarcoma), contrast enhancement margins were most similar (having the highest JSM) to both histological margins than other sequences, with surgical margins having the most similarity. These similarities were not significantly higher than those of other sequences, but low case numbers likely impacted the power of our analyses, and future studies using large case numbers could help confirm these preliminary results. Intra‐tumoral contrast enhancement is a result of neoangiogenesis. These chaotically formed vascular beds contain arteriovenous fistulas and multiple fenestrated or discontinuous capillaries and venules, which results in a high level of vascular permeability.^22^ Both meningiomas and histiocytic sarcomas show similar amounts of contrast enhancement, and this imaging feature consistently has not been a feature that can differentiate these 2 tumor types.^23^, ^24^ In our study, contrast enhancement was most similar (had the highest JSM) to surgical margins in both tumor types, suggesting that this contrast enhancement was associated with solid tumor, tumor infiltration and sites of peritumoral inflammation. We found that FLAIR and T2‐weighted sequences had the least similarity to both solid tumor and surgical margins when compared to contrast enhancement. This result is likely because of the common occurrence of peritumoral edema in both histiocytic sarcoma and meningioma.^8^, ^23^, ^24^ In veterinary neuro‐oncology, radiation planning for meningioma treatment is most commonly performed using margins delineated by iodinated contrast enhancement on CT examinations. Despite the inherent differences in signal production and mechanism of contrast enhancement between iodinated contrast CT and gadolinium contrast MRI, they are comparable techniques^25^ and our results support the continual use of iodinated contrast CT for radiation planning purposes.

A key component of our study was the accurate registration of histology to MRI images. This step brought many challenges because of tissue shrinkage and deformation inherent to the process of brain removal, tissue sectioning, and histology processing involved in creating the histology slides.^26^, ^27^ As a result, the brain tissue on the histology slides was decreased in volume, had an altered contour and shape, and exhibited multiple artifacts including folding and tearing. The process of matching MRI images to histology has been extensively explored in prospective studies using pre‐clinical models and successful protocols have been described.^26^, ^27^, ^28^ However, these protocols rely on the prospective nature of studies requiring multiple planned steps including ex vivo MRI imaging, transcardial paraformaldehyde perfusion for brain fixation, semi‐automated brain extraction, block face photography, 3‐dimensional bloc face reconstruction and 2‐dimensional warping of MR and histology images.^26^, ^28^, ^29^ These methods allow for accurate registration of histology slides to MR images, but could not be applied in our study because of its retrospective nature and the fact that we only had the final histology slides and no block face imaging or ex vivo MRI data. We explored multiple registration options to assist in the matching of our histology and MRI images. Automated registration techniques, although often highly effective, could not be used because we did not want any tumoral tissue to be used in the registration algorithm. If tumoral tissue was included into the registration algorithm it would have biased our results by warping tumor margins and signal intensities to each other. As such, we chose to use the nonlinear warping technique of control point registration This technique allowed us to place points on specific normal anatomic features present on both MR images and the histology scanned image. For example, points were placed on the peripheral margin of the gray matter, within visible sulci, on the margin of the ventricle or within normal white matter features. Doing so allowed us to avoid registering tumor margins to the MR image, which would have biased our results.

After registration of our images, each was evaluated to see how closely the anatomy matched between histology and MRI. We found that, although the final warped histology image was often much closer to the MRI than the original, they often did not match perfectly. This outcome was likely caused by registration error, but also associated with slight differences in the orientation of transverse slices and the impact of different slice thicknesses between methods. Our results should be viewed with these limitations in mind. For example, we are not able to definitively conclude which signal intensity alteration conformed exactly to tumor margins. However, because any errors in registration were inherent to all sequences, we can identify which sequences conformed closest to histology margins when compared to others. In addition, we cannot definitively confirm where surgical and solid tumor margins lie, and it remains possible that tumor cells extended beyond signal intensity changes as has been identified in gliomas in humans using in vivo biopsy techniques.^11^, ^12^, ^13^

Our preliminary study aimed to explore the area of direct comparison of histopathology and MRI, a limited field in the exploration of spontaneous brain disease. To provide the closest correlation between imaging and histopathology, we made our inclusion criteria highly specific and requiring a short imaging to histopathology interval. However, despite attempting to have as short a time interval as possible, our study did include cases with intervals >3 days. These dogs were treated with medications to decrease cerebral edema and inflammation in the brain (information included in Table S1). Although in most cases MRIs were obtained after mannitol infusion, these medications could have impacted the amount of edema observed at necropsy when compared to imaging. Because of the availability of histopathology sections, we only evaluated histopathology and MRI slices and either 1 or 2 levels for each case. Being able to evaluate tumors at more levels is likely to provide a more representative evaluation of the tumor margins, and future work should consider including this approach.

## 5 CONCLUSION

In our study, histology brain sections were registered back to in vivo MRI images of spontaneous tumors in a cohort of dogs so as to directly compare histological margins to signal intensity changes. We determined that contrast enhancement margins were most similar to surgical margins for extra‐axial tumors (meningioma and histiocytic sarcoma) whereas contrast enhancement markedly underestimated margins of gliomas, in which T2‐weighted hyperintensity margins were most similar to surgical margins. Our findings support the continued use of contrast enhancement for surgical and radiation planning purposes in dogs with extra‐axial tumors, but not for intra‐axial gliomas, in which fluid‐rich tissues, that would appear hypoattenuating on CT, should be utilized for planning purposes.

## CONFLICT OF INTEREST

The authors declare no conflict of interest.

## OFF‐LABEL ANTIMICROBIAL DECLARATION

The authors declare no off‐label use of antimicrobials.

## INSTITUTIONAL ANIMAL CARE AND USE COMMITTEE (IACUC) OR OTHER APPROVAL DECLARATION

The authors declare no IACUC or other approval was needed.

## HUMAN ETHICS APPROVAL DECLARATION

The authors declare human ethics approval was not needed for this study.

## Supporting information


**Table S1** Documents the signalment (sex, weight and age), interval between MRI and necropsy, number of slides evaluated, confirmed tumor type and the edema reduction medication received for each included subject.
