# Supplementary material for: Relationship between histological tumor margins and magnetic resonance imaging signal intensities in brain neoplasia of dogs
Source: J Vet Intern Med. 2022 Apr 30;36(3):1039–48. doi: 10.1111/jvim.16431 (PMC9151476; doi:10.1111/jvim.16431)
Supplement: Supplementary file 1 — Table S1 Documents the signalment (sex, weight and age), interval between MRI and necropsy, number of slides evaluated, confirmed tumor type and the edema reduction medication received for each included subject. [file JVIM-36-1039-s001.pdf]

Number	Breed	Sex	Weight (kg)	Age (yrs)	MRI-Necropsy Interval (days)	Number of Slides	Tumor Type	Edema Reduction Medication
1	Chihuahua	MC	3.1	8	7	2	Histiocytic sarcoma, primary CNS	
2	Standard Poodle	MC	15.6	8.6	1	1	Histiocytic sarcoma, multicentric	Mannitol
3	Boston Terrier	FS	8.1	7.7	1	1	Oligodendroglioma, high-grade	Mannitol
4	Borzoi	FI	42	7.4	4	2	Undefined glioma, high-grade	Mannitol
5	Rottweiler	FS	45	4.5	1	2	Histiocytic sarcoma, multicentric	Mannitol
6	French Bulldog	MC	15	8.25	1	2	Oligodendroglioma, high-grade	Mannitol
7	Papillon	FS	3.6	8	0	2	Meningioma, transitional	Mannitol Dexamethasone-SP
8	Boston Terrier	FS	5.3	9.7	0	1	Oligodendroglioma, high-grade	Prednisolone
9	Staffordshire Bull Terrier	MC	33	6.2	1	1	Meningioma, transitional with atypical features	
10	English Setter	FS	20	7.75	3	1	Histiocytic sarcoma, multicentric	Mannitol Prednisolone
11	Labrador Retriever	FS	29.2	11.5	1	1	Meningioma, transitional	Mannitol
12	Boxer	MC	37.7	8.25	3	1	Oligodendroglioma, high-grade	Mannitol Prednisolone

Supplementary Table 1: Documents the signalment (sex, weight and age), interval between MRI and necropsy, number of slides evaluated, confirmed tumor type and the edema reduction medication received for each included subject. For the medication, mannitol was administered as an infusion (0.5-1g/kg IV) 30 minutes before MRI and prednisolone was administered daily (0.5 - 1 mg/kg/day) for 0-7 days.
